# Comparative Cardiac Magnetic Resonance-Based Feature Tracking and Deep-Learning Strain Assessment in Patients Hospitalized for Acute Myocarditis

**DOI:** 10.3390/jcm12031113

**Published:** 2023-01-31

**Authors:** Javier Urmeneta Ulloa, Vicente Martínez de Vega, Ana Álvarez Vázquez, Cristina Andreu-Vázquez, Israel John Thuissard-Vasallo, Manuel Recio Rodríguez, Gonzalo Pizarro, José Ángel Cabrera

**Affiliations:** 1 Cardiology Department, Hospital Universitario Quirónsalud, 28223 Madrid, Spain; 2 Radiology Department, Hospital Universitario Quirónsalud, 28223 Madrid, Spain; 3 Department of Medicine, Faculty of Biomedical and Health Sciences, European University of Madrid, 28670 Madrid, Spain

**Keywords:** feature tracking, deep-learning strain, cardiac magnetic resonance, myocarditis

## Abstract

This study sought to examine the correlation between left ventricular (LV) myocardial feature tracking (FT) and deep learning-based strain (DLS) analysis in the diagnostic (CMRd) and follow-up (CMRf) cardiac magnetic resonance imaging of patients with acute myocarditis. The retrospective study included 17 patients with acute myocarditis and 20 healthy controls. The CMRd took place within 14 days of symptom onset, while the CMRf took place at least 2 months after the event. The global-circumferential FT (FTc) and global-circumferential DLS (DLSc) were analyzed. The continuous variables were compared using paired *t*-tests or the Wilcoxon test, whereas Pearson’s test or Spearman’s test was used to evaluate the correlation between the continuous variables. The time between the CMRd and CMRf was 5 months [3–11]. The LV ejection fraction (LVEF) was 55 ± 6 and 59 ± 4%, p = 0.008, respectively, and 94.1% of the patients showed late gadolinium enhancement (LGE) and myocardial edema on the CMRd. Significantly lower FTc (−16.1 ± 2.2% vs. −18.9 ± 1.9%, *p* = 0.001) and DLSc (−38.1 ± 5.2% vs. −41.3 ± 4.5%, *p* = 0.015) were observed with respect to the controls. Significant increases in the FTc (−16.1 ± 2.2 vs. −17.5 ± 1.9%, *p* = 0.016) and DLSc (−38.1 ± 5.2 vs. −39.8 ± 3.9%, *p* = 0.049) were found between the CMRd and CMRf, which were unrelated to the LGE. The LVEF correlated well with the FTc (r = 0.840) and DLSc (r = 0.760). Both techniques had excellent reproducibility, with high intra- (FTc = 0.980, DLSc = 1.000) and inter-observer (FTc = 0.970, DLSc = 0.980) correlation. There was correlation between the LV DLSc/FTc and LVEF in the patients with acute myocarditis according to the CMRd and CMRf.

## 1. Introduction

Myocarditis is an inflammatory disease of the heart muscle that involves multiple etiological factors, including autoimmune disorders, infections (viruses, bacteria, and fungi, among others), and cardiotoxic drugs/toxins [1]. Its diagnosis remains challenging for clinicians due to the broad spectrum of signs/symptoms at presentation. In recent decades, cardiac magnetic resonance imaging (CMR) has emerged as the main non-invasive tool for evaluating myocardial impairment in the diagnosis of myocarditis, as endorsed by the European Society of Cardiology in its latest guidelines [2,3].

The strength of CMR lies in its excellent capacity for tissue characterization and cardiac functional assessment, which permits the determination of the presence/absence of the previously well-established modified Lake Louise Criteria [4] through sequences aimed at detecting edema (T_2_-STIR and T_2_-map) and myocardial fibrosis (focal in the presence of late gadolinium enhancement [LGE]; diffuse in T_1_-mapping sequences).

Recently, the analysis of myocardial deformation by means of feature tracking (FT, analogous to speckle tracking in echocardiography for the longitudinal strain) has become possible using the basic cine sequences acquired in CMR studies, and this technique has proven useful in the assessment of subclinical cardiac pathology [5,6]. While several studies have assessed the applicability of FT in the field of myocarditis, the results have been contradictory [7,8,9], and to the best of our knowledge, only two studies have evaluated the clinical application of FT using follow-up MRI scans in patients after an acute myocarditis episode [7,8]. Very recently, myocardial deformation analysis based on deep-learning strain (DLS) quantification and inferring the 2D velocity field from the series of short-axis steady-state free-precession (SSFP) cine images has become feasible. This is a very novel technique that has recently been introduced into clinical research.

The aim of the present study was to evaluate the applicability of the FT and DLS methods for the CMR of patients hospitalized for acute myocarditis with clinical/analytical expression/diagnostic CMR (CMRd) and its evolution in follow-up CMR (CMRf). We compared the findings obtained using the two advanced deformation analysis techniques with the CMR available in our center.

## 2. Materials and Methods

### 2.1. Study Population

**Figure 1:**
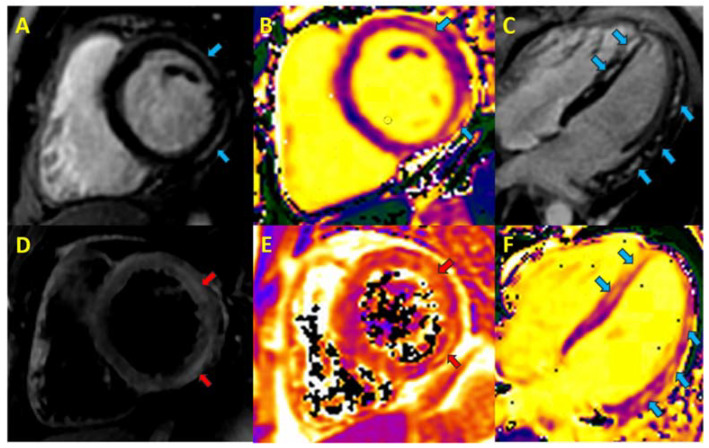
Cardiac magnetic resonance in acute myocarditis. Blue arrows, focal fibrosis; red arrows, myocardial oedema **A**. Phase-sensitive inversion recovery (PSIR) short axis, lateral subepicardial late gadolinium enhancement (LGE). **B**. T_1_ short-axis map. **C**. PSIR 4-chamber long axis, non-ischemic septal and lateral LGE. **D**. T_2_-short tau inversion recovery short-axis, foci of myocardial edema. **E**. T_2_-map, myocardial oedema. **F**. T_1_-long-axis 4-chamber map.

The research protocol for this retrospective observational study was approved by the ethics committee of the national reference center (nº14/22). We included patients with a diagnosis of acute myocarditis who were hospitalized between 2016 and 2021 and who had CMRf performed during follow-up at least 2 months after the event [8,10]. All the included patients had clinical chest pain/fever and elevated cardiac biomarkers on admission. All the patients had to fulfil the modified Lake Louise Criteria (myocardial edema and fibrosis) for a diagnosis of myocarditis in the CMRd performed during the acute phase of the disease (Figure 1), i.e., within 14 days of symptoms onset [11].

The exclusion criteria included cardiomyopathy and a previous history of ischemic or valvular heart disease, as well as the general contraindications for CMR. The baseline characteristics of the patients were extracted from their medical records. All the patients gave prior consent for CMR and the use of their imaging data for educational/scientific purposes. No external funding was received for any aspect of this work.

### 2.2. Acquisition and Post-Processing of CMR Imaging

The imaging studies were performed on a 1.5T GE Optima MR450w (GE Healthcare, Milwaukee, WI, USA) MRI unit using a 32-channel multi-element surface antenna and electrocardiographic synchronization. The cine images were obtained in expiratory apnea using the conventional SSFP sequences in 4-, 3-, and 2-chamber longitudinal slices, and in 10–15 contiguous short-axis slices covering both ventricles from the base to the apex. Approximately 8–10 min after the intravenous infusion of 0.15 mmol/kg gadobutrol (Gadovist^®^, Bayer Schering Pharma AG, Berlin, Germany; 1 mmol/mL), the LGE images were acquired, with the same planning as used for the cine images, using a T_1_-weighted inversion recovery (IR) gradient-echo T_1_-weighted sequence.

For the assessment of the edema, we used both the classic T_2_-weighted sequences and short tau inversion recovery (STIR) sequences, which were obtained prior to the gadolinium administration, in the short axis from the base to the apex, as well as the T_2_-map sequences in the end-diastole in the basal, middle, and apical cut in the short axis, and a longitudinal cut in 4 chambers, using the T_2_-weighted TSE (turbo spin echo) sequences with different T_2_ times (11, 37, 63, and 90 msec), with the repetition time (TR = 1 × R-R). The T_1_-mapping was performed with a modified Look-Locker IR sequence using a 3(3)5 scheme in the same four planes acquired for the T_2_-map prior to and 15 min after the contrast infusion.

All the CMR studies were analyzed by a cardiologist (*) (EACVI European CMR level 3 accreditation) and a radiologist (**) with CMR experience (>6 and >9 years, respectively). The cardiac functional analysis was performed retrospectively using the dedicated advanced analysis software CVI42 (v.5.13.8; Circle Cardiovascular Imaging Inc., Calgary, AB, Canada). The left (LVEF) and right (RVEF) ventricular ejection fractions were measured from the cine sequences using the disc summation method. The myocardial edema on the T_2_-STIR sequences was visually assessed and with agreement between the two experts, whereas the mapping analysis was performed by tracing a region of interest (ROI) in the septum on each of the acquired slices. The LGE in each segment was visually classified as subepicardial, intramyocardial, subendocardial, transmural, or non-enhancement. The endocardial and epicardial contours in the LGE short-axis sequences were manually traced, and the percentage of enhanced mass volume was calculated using the full width at half maximum (FWHM) method.

The feature tracking was performed using the advanced cardiac analysis software CVI42 (v.5.13.8; Circle Cardiovascular Imaging). The global circumferential FT (FTc) was performed on the short-axis, 4-, 3-, and 2-chamber views in expiratory apnea using the cine SSFP sequences. For this purpose, the contours were traced along the LV endocardial and epicardial border in both the end-diastole and end-systole in all the basal slices (except the two–three most basal slices with the highest percentage of atrial wall and with less than 50% myocardial wall) and in a 4-, 3-, and 2-chamber reference slice. The contours subsequently propagated automatically through all the phases, and in the case of an erroneous propagation, it was manually edited in the respective slice, again propagating automatically. The FTc was obtained from the derivative of the tracking of the features within the myocardium (defined by the inputted endocardium and epicardium contours) throughout the cardiac cycle, resulting in an overall FTc value.

All the DLS analyses were performed using the advanced research-use-only strain feature hosted on the Arterys platform (Arterys Inc., San Francisco, CA, USA) and developed by the AiDA Laboratory. The model was based on artificial intelligence or deep learning, and it inferred the 2D myocardial velocity fields from the short-axis cine SSFP image series. These velocity fields were then used to calculate the pixel-wise myocardial strain rate and strain, thereby providing the regional circumferential and radial strain measurements. The algorithm was trained using the coregistered 4D Flow and short-axis cine SSFP images acquired during the same examination for each patient with full left-ventricle coverage. Once the LV endocardial and epicardial border at the end-diastole and end-systole, as well as the RV inferior–anterior septal junction points, had been traced, a mathematical algorithm automatically calculated the myocardial deformation values for each frame of the cardiac cycle, obtaining among them the 2D global circumferential DLS (DLSc). It is not possible to calculate the global longitudinal DLS using this technology at the present time. Finally, the FTc and DLSc results between the CRMd and CRMf were compared.

### 2.3. Statistical Analysis

The descriptive statistics are presented as the absolute (n) and relative (%) frequency for the categorical variables and as the mean ± standard deviation (SD) or median (interquartile range, IQR) for the continuous variables, depending on the parametric or non-parametric behavior of the variables, respectively. The continuous variables were compared using Student’s paired *t*-test or the Wilcoxon test, while the categorical variables were analyzed using McNemar’s test. Pearson’s or Spearman’s correlation was used to evaluate the correlation between the continuous variables, after checking the normality of the variables. The absolute intraclass correlation coefficient (ICC) values were calculated to determine the intra- and inter-observer reliability. Receiver operating characteristic (ROC) plots were used to determine the FTc and DLSc values that diagnosed circumferential myocardial deformation involvement with better specificity and sensitivity, and the area under the curve (AUC) and its 95% confidence interval (CI) was calculated. The data analysis was performed using SPSS, version 25.0 (IBM Corp., Armonk, NY, USA) at a 5% significance level.

## 3. Results

### 3.1. Study Population

A total of 17 patients with acute myocarditis and 20 healthy individuals were included in this study. The baseline characteristics of the patients in the study are shown in Table 1. The CT angiography was normal in 4/17 (24%) patients, while the cardiac catheterization was normal in 6/17 (35%) patients, without significant lesions in the coronary arteries. The mean age of the patients was 37 ± 17 years, 88.2% were male, and 11.8% had hypertension or previous dyslipidemia. All the patients had clinical manifestations on admission, as characterized by fever and chest pain in 52.9% of patients, and by chest pain alone in the remaining 47.1%. ST-segment repolarization abnormalities were the most common presentation (64.7%) on the electrocardiogram. Regarding treatment, 41.2% received beta-blockers and 47% received angiotensin-converting enzyme (ACE) inhibitors at hospital discharge. The mean age of the healthy group was 44 ± 10 years (35% male), 10% were hypertensive, and 30% had previous dyslipidemia. CMR was mostly performed for palpitations in this group, with a normal echocardiography and ECG.

### 3.2. Cardiac Magnetic Resonance Imaging

The morphofunctional data from the CMR findings are shown in Table 2. The time between the CMRd and CMRf was 5 months [3,4,5,6,7,8,9,10,11]. The LVEF and RVEF were preserved in the majority of patients in both the CMRd (LVEF = 55 ± 6%; RVEF = 55 ± 6%) and CMRf (RVEF = 59 ± 4%; RVEF = 57 ± 4%). LGE was observed in 94.1% (16/17) of the patients, with a lateral subepicardial location being the most frequent distribution (41.2%), followed by a lateral/septal subepicardial pattern (23.5%), a subepicardial ring pattern (17.7%), and subepicardial septal involvement (11.8%). The percentage of LGE on the CMRc was 8% (5–12%), although it was significantly lower on the CMRf (3% [2–5]%, p = 0.001). None of the CMRd images showed an ischemic pattern of LGE. The CMR in the healthy group showed preserved LVEF (63 ± 5%) and RVEF (62 ± 6%) values.

Regarding the quantitative map values, there was a normalization of the values between the CMRd and CMRf for the native myocardial T_1_ (1083 ± 72 vs. 976 ± 20 msec, *p* = 0.008), T_2_ map (61 [57–68] vs. 49 [47–51] msec, *p* = 0.003), and extracellular volume (ECV) (30 ± 2 vs. 25 ± 2%, *p* < 0.001). In total, 94.1% (16/17) of patients presented with signal hyperintensity in the T_2_-STIR sequences in the regions with LGE compatible with myocardial edema on the CMRd. Only two patients (11.8%) presented with segmental contractile alteration, such as septal hypokinesia, on the CMRd.

**Figure 2:**
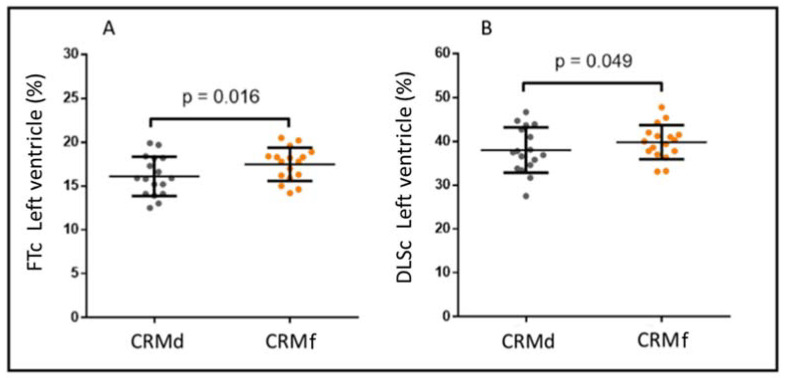
Analysis of the global circumferential feature tracking (FTc) (**A**) and global circumferential deep learning-based strain (DLSc) (**B**) in the diagnostic (CMRd) and follow-up (CMRf) cardiac magnetic resonance imaging.

**Figure 3:**
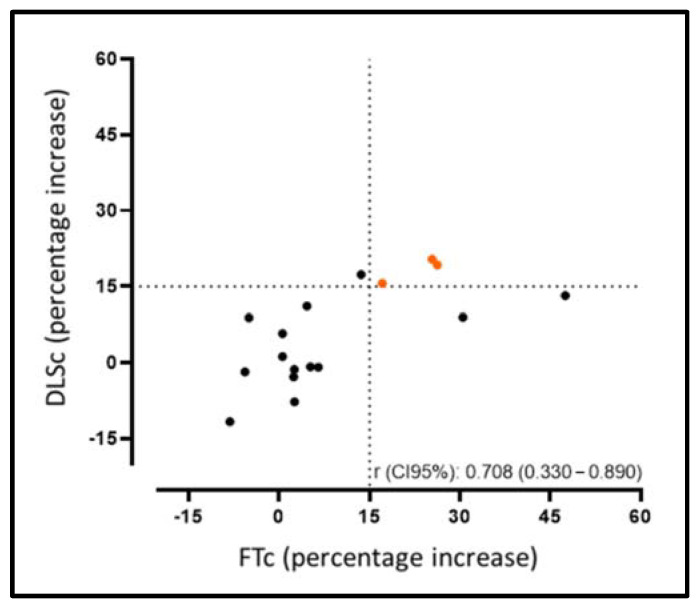
Correlation agreement rate for the improvement between the FTc and DLSc, increasing by >15% of their relative value from the CMRd to CMRf.

As shown in Figure 2, we observed an increase in the FTc (−16.1 ± 2.2% vs. −17.5 ± 1.9%, *p* = 0.016) and DLSc (−38.1 ± 5.2% vs. −39.8 ± 3.9%, *p* = 0.049) values between the CMRd and CMRf, increasing by >15% of their relative value in 29% (5/17) (FTc) and 25% (4/17) (DLSc) of the patients, respectively. At the same time, we found good correlation between the techniques in those patients in whom there was no increase in the FTc/DLSc (r = 0.70; *p* = 0.002). Our results show that those patients in whom there was an improvement in the strain values according to the DLSc technique also showed an improvement according to the FTc technique, meaning they were equally reproducible in the opposite case (Figure 3) (Supplementary Figures S1 and S2, Video S1–S4).

In our sample, the FTc/DLSc improvement was not related to the evolution of the percentage of LGE between the CMRd and CMRf (FTc *p* = 0.874; DLSc *p* = 0.740).

The patients with myocarditis had significantly lower FTc/DLSc values than the healthy group: FTc −16.1 ± 2.2% vs. −18.9 ± 1.9% (*p* = 0.001) and DLSc −38.1 ± 5.2% vs. −41.3 ± 4.5% (*p* = 0.015). The ROC curves determined that values of −17% for the FTc (AUC 0.816 95% CI: 0.671–0.962; S = 0.95; E = 0.65) and −38% for the DLSc (AUC 0.735 95% CI: 0.551–0.919; S = 0.90; E = 0.65) were the best cut-off points for diagnosing circumferential myocardial deformation involvement in this setting in our population (Supplementary Figure S3). Of note, we found good correlation between the LVEF-FTc (r = 0.84) and LVEF-DLSc (r = 0.76) (Supplementary Figure S4).

The strain assessment by means of CMR in our sample showed excellent intra-observer (DLSc: ICC = 1.00, 95% CI: 0.99–1.00; FTc: ICC = 0.98, 95% CI: 0.94–0.99) and inter-observer (DLSc: ICC = 0.98, 95% CI: 0.95–0.99; FTc: ICC = 0.97, 95% CI: 0.90–0.99) variability with both techniques.

## 4. Discussion

The main findings of our study are the following. We found excellent reproducibility with high intra-/inter-observer correlation for the DLSc and FTc. In addition, the DLSc/FTc values were lower in the patients with acute myocarditis than in the healthy group, and there was good correlation between the DLSc and FTc findings and the LVEF. Finally, no relationship was found between the LGE grade and DLSc/FTc-based improvement in our population.

Previously, to the best of our knowledge, only two groups have assessed patients after admission for acute myocarditis using CMR-FT follow-up. In the first of these, Luetkens et al. [7] observed an improvement in the FT values at follow-up. They also established the presence of edema in the initial study as a prognostic factor for recovery in the CMRf study, and they identified LGE (66% in their sample) as a predictor of events in these patients. Additionally, they found that only longitudinal FT could discriminate the recovery of ventricular function after myocarditis, with a cut-off point of −16.9% (below which the probability of recovery was lower) and with a lower value for those with reduced LVEF (−12.3%). In line with our findings, Secchi et al. [8] highlighted the value of FTc in patients with acute myocarditis, finding lower values in the initial/follow-up study compared with the control group. At the same time, they showed correlation between the FTc, the presence of edema (acute phase), and LGE.

In agreement [8,12,13,14,15,16] and in divergence [7,17,18,19] with other groups, we consider the circumferential strain to be the more robust and reproducible tool when compared with longitudinal strain measurements by means of CMR. Similarly, considering that there is generally no subendocardial involvement in myocarditis [11], it is highly likely that a more accurate assessment of myocardial deformation in this scenario is obtained via the circumferential strain by assessing the myocardial fibers affecting the epi-/intra-myocardial region. Accordingly, we considered only the circumferential strain in the analysis. On the other hand, this is virtually the first study that has evaluated the DLS in a clinical scenario. Along the same lines, Porcari et al. [11] assessed the FTc in patients with a confirmed and suspected diagnosis of myocarditis, finding a reduction in the biventricular FT values in both groups compared with the control group; in this case, with no correlation with the presence of edema or LGE in these patients. At the same time, they demonstrated the low inter-observer variability and high reproducibility of the technique in this scenario. Using the DLS and FT techniques for the CMRd and CMRf, we also found excellent inter-observer variability (DLSc = 0.98; FTc = 0.97) independently of the software used (with a minimal difference between them, which could be explained by the different post-processing methods), provided that the study is performed using the same analysis technique during the diagnostic and follow-up studies.

Among the aspects to be highlighted, we would mention that despite representing different strain values for the DLSc than for the FTc (different analysis techniques), we obtained good correlation between the two strain values in the CMRd and CMRf, showing a relative increase of more than 15% [20] with both techniques.

While we have to consider that our sample was composed of patients who met the strict criteria for a diagnosis of acute myocarditis at the time of hospital admission (94.1% with the presence of edema in the STIR and LGE sequences on the baseline CMR and elevated troponin T), we did not find correlation between having a higher percentage of LGE and presenting with worse FTc/DLSc values in the CMRf, with the caveat that we cannot assess the direct correlation between the presence/absence of LGE and the DLSc/FTc strain values. Likewise, we did not find correlation between the evolution of the degree of LGE between the CMRd and CMRf and the improvement of strain by both techniques (FTc/DLSc).

Regarding the relationship of edema and LGE with FT, there are also contradictions according to previous studies. In a study sample where 77% of the patients had LGE and 75% showed myocardial edema in CMR, Luetkens et al. [19] found that the alteration in the longitudinal FT was the most associated parameter with the presence of myocardial edema. Contrastingly, Chen et al. [14] found only correlation between decreased values of FTc and the presence of LGE, without finding differences with respect to the longitudinal FT. The higher the LGE, the worse the LVEF and the lower the FTc values of the patients. We found no significant association between these variables, likely in view of the high percentage of patients with edema/LGE in our sample.

Several groups have highlighted the importance of determining whether or not the technique has added value in patients with myocarditis and preserved the LVEF, with the impact on the subclinical evaluation of these patients that this would imply. Studies such as that by Gatti et al. [9] using an LVEF cut-off value of 55% found no differences in the circumferential, radial, or longitudinal FT values in 30 patients with a diagnosis of myocarditis and preserved LVEF as compared with the control group. Conflictingly, in a group with clinical suspicion of myocarditis, including patients with preserved LVEF (>55%) undergoing non-contrast CMR, Ravesh et al. [12] found a high negative predictive value (87.5%) of the FTc in this scenario. Likewise, Baeßler et al. [21] found reduced circumferential/longitudinal FT values in patients with a confirmed diagnosis and suspected myocarditis compared with the control group in those with an LVEF <55%, and they also established a direct relationship between the two in which the patients with preserved LVEF (>55%) did not show a decrease in the FT values. In our study, we found good correlation between the LVEF-FTc (r = 0.84) and LVEF-DLSc (r = 0.76); thus, the higher the LVEF, the better the FTc and DLSc values in our patients.

To the best of our knowledge, this is the first study in patients with a follow-up CMR after hospitalization for acute myocarditis that performed a comparative analysis of myocardial deformation simultaneously between the FTc and DLSc techniques. Our findings reflect a good concordance of results between the two analysis techniques despite the disparity between their values, showing a decrease in both with respect to the control group and the good correlation of both techniques with the LVEF, with excellent intra- and inter-observer variability as long as the same tool was used for the follow-up.

### Study Strengths and Limitations

Our study has several limitations that should be considered. First of all, it is a single-center retrospective study with a limited number of patients. However, considering the strict inclusion criteria for our sample (94.1% with myocardial edema/LGE, elevated cardiac biomarkers, hospital admission, CMRf at least 2 months after the event), we consider that it was adequate for the preliminary analyses, which will hopefully open the door for future multicenter studies. Nevertheless, given the relatively small number of patients, the generalizability of our cut-off points for diagnosing circumferential myocardial deformation involvement should be treated with caution. On the other hand, there is a gender difference between the normal and myocarditis patients in our sample that should be taken into consideration. Some authors, however, have shown that there are no significant differences between the circumferential strain values by gender [22]. We considered only the circumferential strain, excluding the longitudinal/radial strain values, in view of our previous experience of the greater reproducibility and applicability of the former. Regional strain values were not considered in view of the variability we observed in the sub-analysis, as in other working groups [9]. We did not perform endomyocardial biopsy in any patient due to the lack of clinical indication (preserved LVEF and favorable evolution) and the risk involved in using this technique.

While we acknowledge that we used two different myocardial deformation analysis techniques in the CMR, the purpose of the study was to assess the correlation between their results, which demonstrated their feasibility and reproducibility as long as the same tool is used during their follow-up.

## 5. Conclusions

The assessment of LV myocardial deformation by means of CMR strain DLSc-FTc in patients with acute myocarditis showed good correlation between their values and with the LVEF in the CMRd and CMRf. Lower DLSc/FTc was observed in this scenario in the CMRd compared with the healthy group, with a subsequent increase in the CMRf, which was unrelated to the degree of LGE in our population.

## Figures and Tables

**Table 1:** Baseline characteristics of the patient study population (n, % unless otherwise stated).

Baseline Characteristics	Myocarditis Patients	Healthy Group
Age (mean ± SD), years	37 ± 17	44 ± 10
Body mass index (Kg/m^2^)	25 ± 4	25 ± 3
Male	15/17 (88.2%)	7/20 (35%)
Hypertension	2/17 (11.8%)	2/20 (10%)
Diabetes	0/17 (0%)	0/20 (0%)
Dyslipidemia	2/17 (11.8%)	6/20 (30%)
Clinical presentation:		
- Asymptomatic	0/17 (0%)	0/20 (0%)
- Fever and chest pain	9/17 (52.9%)	0/20 (0%)
- Chest pain	8/17 (47.1%)	
ECG:		
- Normal	1/17 (5.9%)	20/20 (100%)
- ST segment alterations	11/17 (64.7%)	
- Q pathological	1/17 (5.9%)	
- T negative	2/17 (11.8%)	
- Right bundle branch block	2/17 (11.8%)	
Analytical parameters:		
- Fibrinogen (mg/dL)	626.3 ± 145.9	N/A
- Ferritin (ng/mL)	160.4 ± 123.5	
- Leukocytes (10^9^/L cells per liter)	8.7 ± 2.4	
- CRP (mg/dL)	0.98 [0.62–4.08]	
- Troponin T (ng/mL)	972 ± 690	
Coronary study:		
- Not performed	7/17 (41.2%)	N/A
- Normal CT angiography	4/17 (23.5%)	
- Normal cardiac catheterization	6/17 (35.3%)	
Treatment:		
- Beta-blockers	7/17 (41.2%)	N/A
- ACE inhibitors	8/17 (47%)	

n, %, unless otherwise indicated.

**Table 2:** Findings of diagnostic and control cardiac magnetic resonance imaging (n, %, unless otherwise indicated).

CMR Findings	CMRd	CRMf	*p*
LGE pattern:			
- Lateral subepicardial	7/17 (41.2%)		
- Septal subepicardial	2/17 (11.8%)	N/A	
- Lateral/septal subepicardial	4/17 (23.5%)		
- Ring-like pattern	3/17 (17.7%)		
LVEF (%)	55 ± 6	59 ± 4	0.008
RVEF (%)	55 ± 6	57 ± 4	0.113
LGE quantification (%)	8 [5–12]	3 [2–5]	0.001
Mapping:			
- T_1_-native myocardial (msec)	1083 ± 72	976 ± 20	0.008
- Extracellular volume (%)	30 ± 2%	25 ± 2%	<0.001
- T_2_-mapping (msec)	61 [57–68]	49 [47–51]	0.003
FTc (%)	−16.1 ± 2.2	−17.5 ± 1.9	0.016
DLSc (%)	−38.1 ± 5.2	−39.8 ± 3.9	0.049

n, %, unless otherwise indicated. CMR = cardiac magnetic resonance; CMRd = diagnostic CMR; CMRf = follow-up CMR; LGE = late gadolinium enhancement; LEVF = left ventricular ejection fraction; RVEF = right ventricular ejection fraction; FTc = global circumferential feature tracking; DLSc = global circumferential deep learning-based strain; N/A = not applicable.

## Data Availability

Data supporting the findings of this study can be found in the article or its supplementary material, and detailed data are available from the corresponding author upon reasonable request.

## Supplementary Materials

The following supporting information can be downloaded at https://www.mdpi.com/article/10.3390/jcm12031113/s1, Figure S1: Evolutionary control by global circumferential feature tracking (FTc) (A and C) and global circumferential deep learning-based strain (DLSc) (B and D) in a patient with acute myocarditis. Normalization of FTc/DLSc during evolution in the same patient, global value. A, B, diagnostic cardiac magnetic resonance imaging. A. FTc of −15.2%. B. DLSc of −34.6%. C, D, follow-up cardiac magnetic resonance imaging. C. FTc of −17.8%. D. DLSc of −40%. Figure S2: Cardiac magnetic resonance imaging control by global circumferential feature tracking (FTc) (A and C) and global circumferential deep learning-based strain (DLSc) (B and D) in patients with acute myocarditis. FTc/DLSc unchanged during evolution in the same patient, global value. A, B, diagnostic cardiac magnetic resonance imaging. A. FTc of −18.2%. B. DLSc of −38.1%. C, D, follow-up cardiac magnetic resonance imaging. C. FTc of −18.3%. D. DLSc of −38.9%. Figure S3: ROC (receiver operating characteristic) plots to determine cut-off values for diagnosing circumferential myocardial deformation. Figure S4: Correlation analysis of LVEF-FTc and LVEF-DLSc in CMRd. Video S1: CRMd. FTc decreased (−15.2%). Video S2: CRMf. FTc improvement (−17.8%). Video S3: CRMd. DLSc decreased (−34.6%). Video S4: CRMf. DLSc improvement (−40%).

## Abbreviations

ACE	Angiotensin-converting enzyme
AUC	Area under the curve
CMRc	Cardiac magnetic resonance, control
CMRf	Cardiac magnetic resonance, follow-up
CT	Computed tomography
DLSc	Circumferential deep learning-based strain
ECV	Extracellular volume
FTc	Circumferential feature tracking
ICC	Intraclass correlation coefficient
IR	Inversion recovery
LGE	Late gadolinium enhancement
LVEF	Left ventricular ejection fraction
RVEF	Right ventricular ejection fraction
ROI	Region of interest
STIR	Short tau inversion recovery
