# Supplementary figures and images for: Comparative Cardiac Magnetic Resonance-Based Feature Tracking and Deep-Learning Strain Assessment in Patients Hospitalized for Acute Myocarditis
Source: J Clin Med. 2023 Jan 31;12(3):1113. doi: 10.3390/jcm12031113 (PMC9917983; doi:10.3390/jcm12031113)

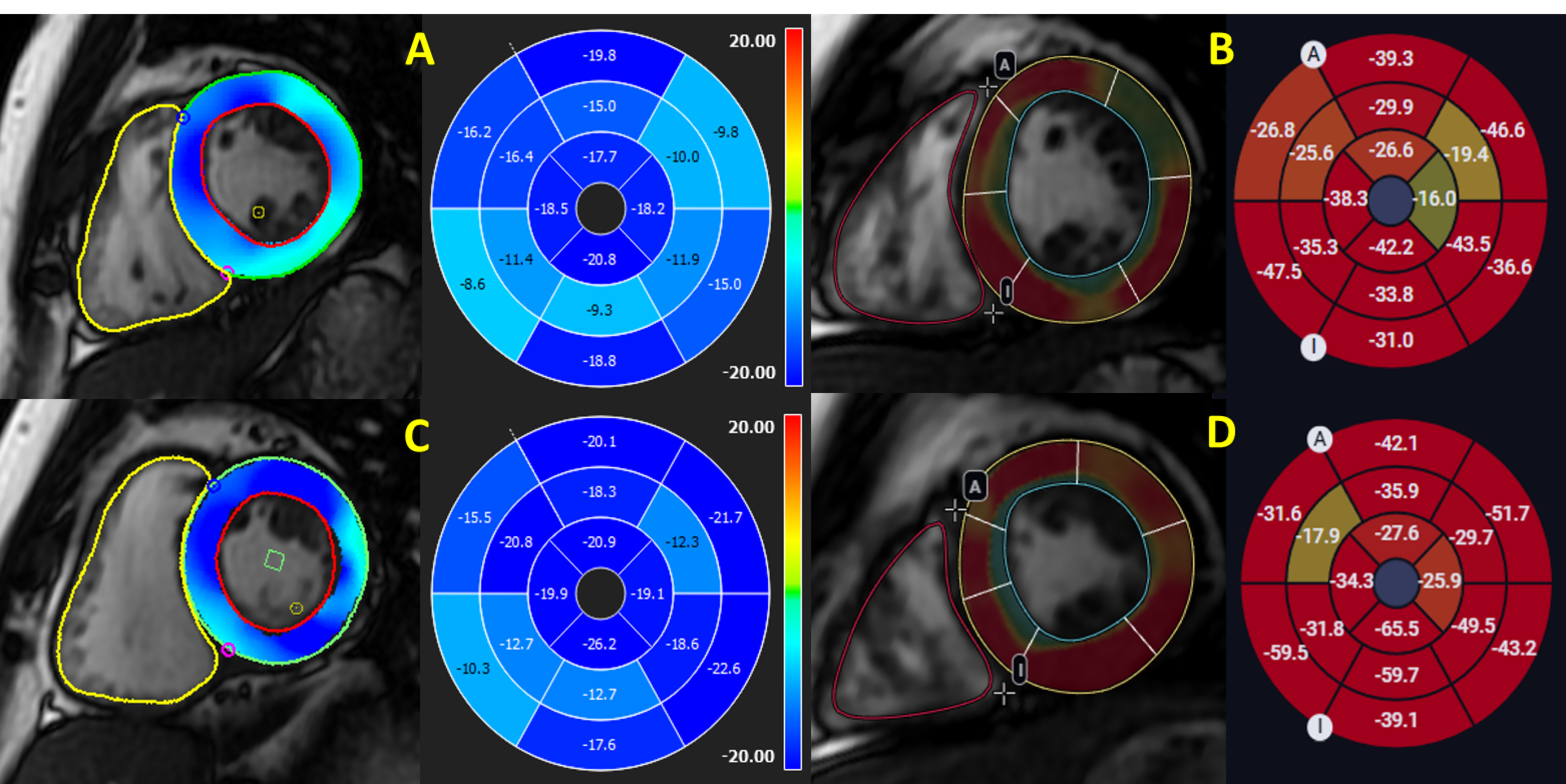

Supplement: Supplementary file 1 [file jcm-12-01113-s001.zip › jcm-2181280-supplementary/Figure S1.tiff]

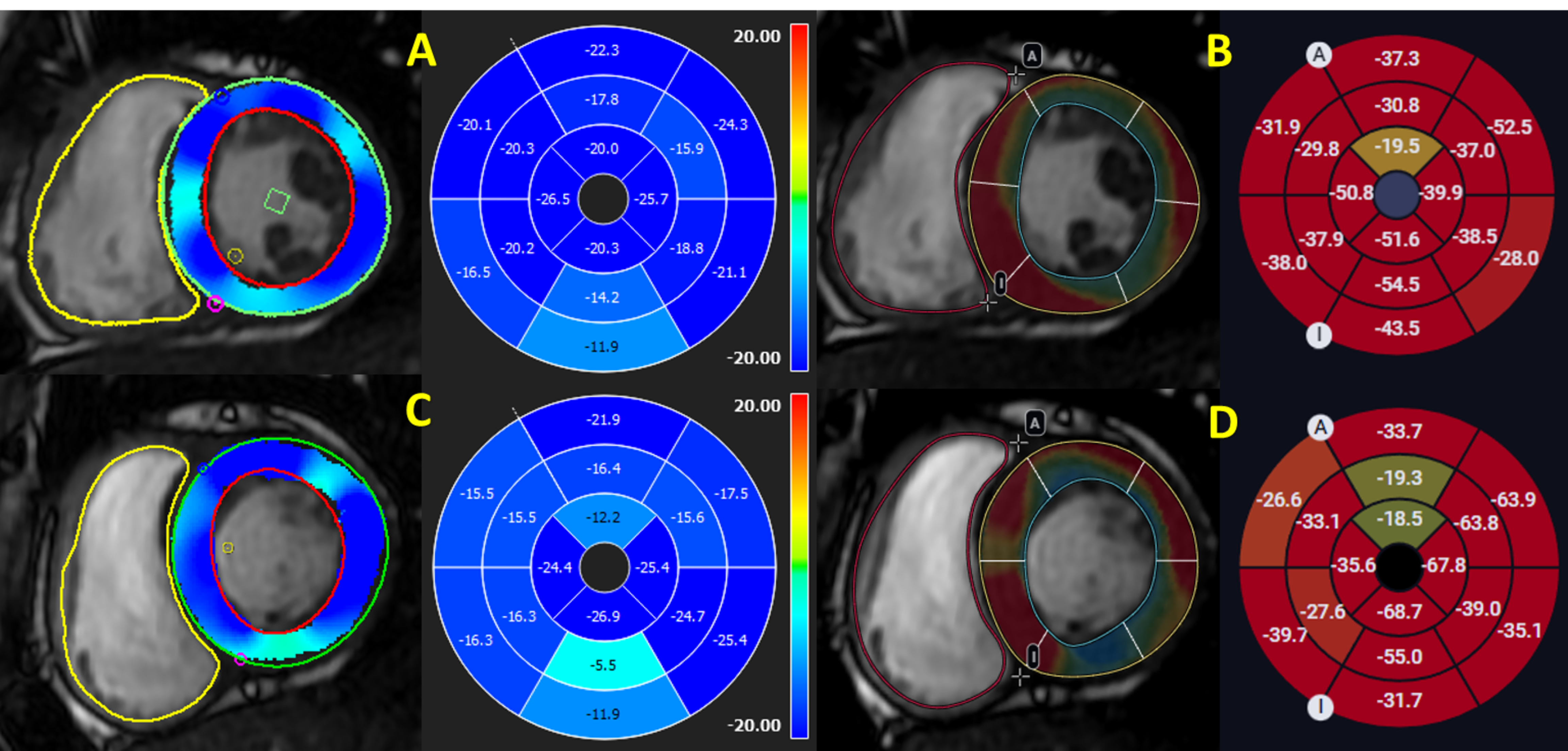

Supplement: Supplementary file 1 [file jcm-12-01113-s001.zip › jcm-2181280-supplementary/Figure S2.tiff]

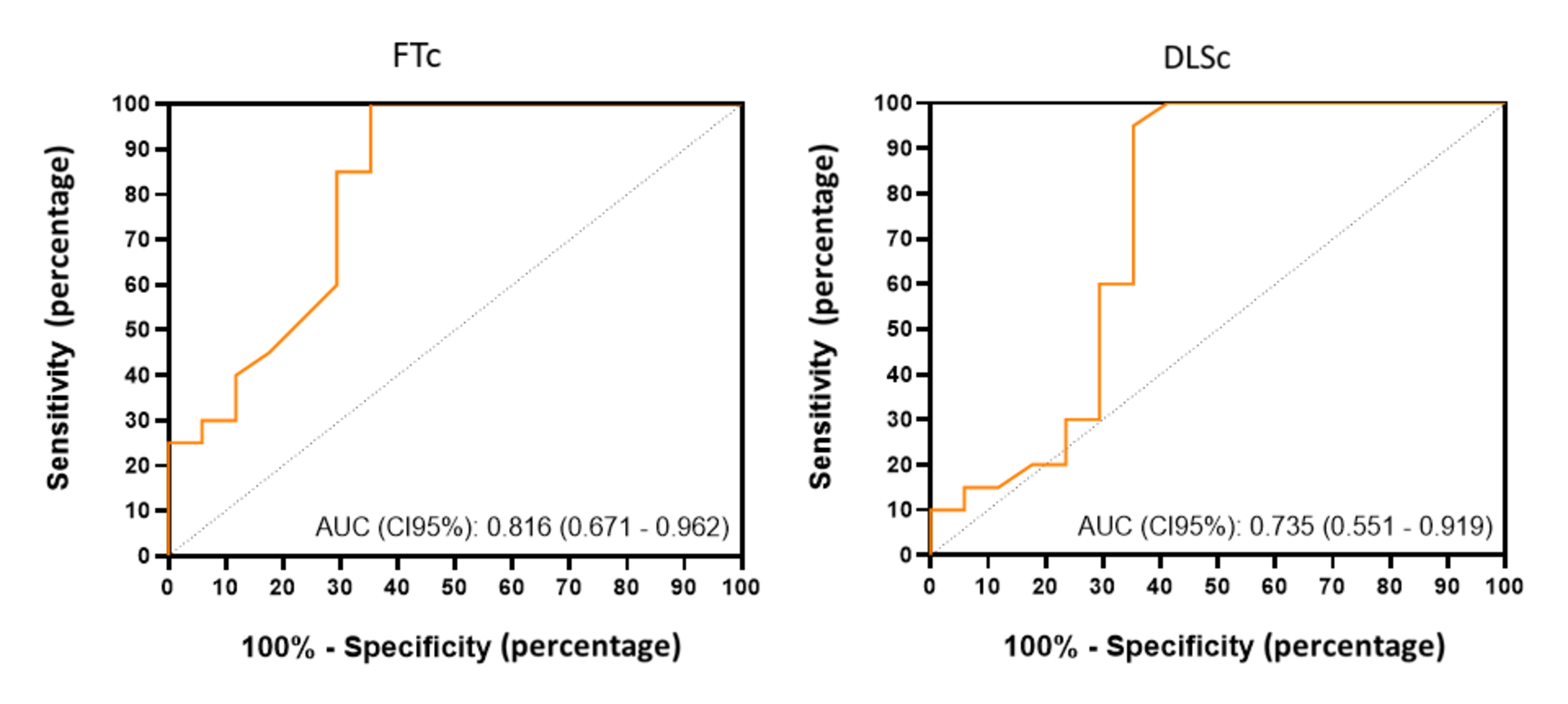

Supplement: Supplementary file 1 [file jcm-12-01113-s001.zip › jcm-2181280-supplementary/Figure S3.tiff]

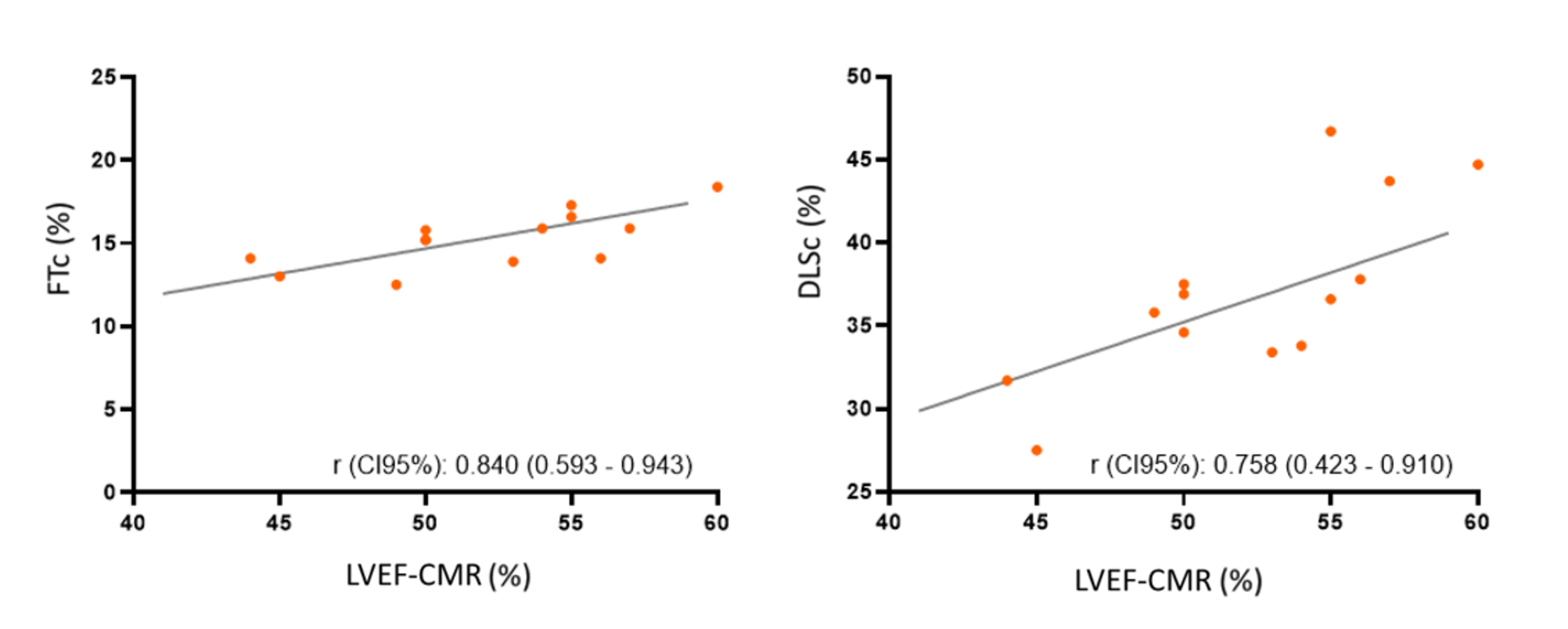

Supplement: Supplementary file 1 [file jcm-12-01113-s001.zip › jcm-2181280-supplementary/Figure S4.tiff]
